# Regulatory effects of differential dietary energy levels on spermatogenesis and sperm motility of yellow-feathered breeder cocks

**DOI:** 10.3389/fvets.2022.964620

**Published:** 2022-09-29

**Authors:** Fuguang Xue, Yifan Liu, Ziyang Lv, Jian Zhang, Shiyuan Xiong, Liqing Zha, Zhiyu Liu, Jingting Shu

**Affiliations:** ^1^Key Laboratory for Poultry Genetics and Breeding of Jiangsu Province, Jiangsu Institute of Poultry Science, Yangzhou, China; ^2^Nanchang Key Laboratory of Animal Health and Safety Production, Jiangxi Agricultural University, Nanchang, China; ^3^College of Economics and Management, Jiangxi Agricultural University, Nanchang, China

**Keywords:** breeder cocks, energy, testis, spermatogenesis, mitochondria

## Abstract

The semen quality of breeder cocks profoundly impacted the numbers of matched layer hens and the economic benefits of the poultry industry. Adequacy and balance of poultry nutrition, especially the energy provision, critically modulated the reproductive potential of breeder cocks, however, the underlying mechanism was still unclear. For the purpose of this study, a total of 90 yellow-feathered 13-week-old roosters with the same age in days and similar body weight (1,437 ± 44.3 g) were selected and randomly divided into the low energy diet (LE), the moderate energy diet (ME), and the high energy diet (HE) treatments. The phenotypic parameters related to reproduction include semen quality, fertility, and hatchability, and the testis morphological parameters, including seminiferous epithelium length (SEL), seminiferous tubule perimeter (STP), seminiferous tubule area (STA), and Johnsen score, were measured to investigate the regulatory effects of different energy diets on reproductive performances. Furthermore, spermatogenesis and sperm motility-related genes, which included the sry-related high mobility group box (*SOX*) gene family and sperm-associated antigen (*SPAG*) gene family, and mitochondria apoptosis-related genes, such as *Cyt-C, Bcl-2*, and *Bax*, were measured to determine the underlying mechanism of energy on the reproductive performances. The The results showed that the gonadosomatic index and sperm motility in the ME treatment significantly increased compared with the LE treatment. Chickens in the ME treatment showed a preferable performance of testis development, especially a significant increment of SEL and Johnsen Score, compared with the LE and HE treatments. Finally, spermatogenesis-related genes, which included *SPAG6, SPAG16, SOX5, SOX6*, and *SOX13*, and apoptosis-related genes of mitochondria, such as the *Cyt-C* and *Bcl-2*, were significantly upregulated in the ME treatment. This study concluded that proper energy provision stimulated regular energy metabolism for spermatogenesis and sperm capacitation, which finally increased semen quality and reproductive performances of breeder cocks.

## Introduction

Reproductive performance, especially the semen quality of breeder cocks profoundly impacted the development of the poultry industry (1, 2). Roosters in high genetically selected poultry strain successfully matched 80–100 layer-hens, whereas a certain number of Chinese native chicken breeds matched only 10 layer-hens because of the lower semen volume ejaculation and dispirited sperm motility (3). Therefore, proper strategies to enhance spermatogenesis and sperm motility of native breeder cocks are increasingly required.

Spermatogenesis is a complex cell differentiation and sperm synthesis bioprocess which mainly includes the mitotic stage, the meiosis stage, and the spermiogenesis stage (4–7). Mature sperm further undergoes sperm capacitation to ensure adequate sperm motility to complete the fertilization in the female reproductive tract (8, 9). A certain number of genes, which mainly included the sry-related high mobility group box (*SOX*) gene family (10) and sperm-associated antigen (*SPAG*) gene family (11, 12), synergistically participated in spermiogenesis and the capacitation process and further determined the semen quality of breeder cocks. All these genes were functionally expressed in testis and were regulated by genetic traits, natural environment management, and nutrition.

Adequacy and balance of poultry nutrition, especially the energy provision, are considered to ensure the achievement of the normal inherent physiological capacity of other nutritional elements during the growth stage of breeder cocks (13–15). Disordered dietary energy significantly interfered with regular energy metabolism, which mainly occurred in the mitochondria in the cell, and further inhibited semen quality (16); however, the underlying mechanism of the effect of mitochondrial energy metabolism on semen quality is still unclear.

The mechanism of energy metabolism occurred in the mitochondria in cells through the electron transport chain (17) to satisfy the energy requirement of both spermatogenesis and the sperm capacitation process. Mitochondria are mainly involved in energy provision during spermatogenesis and the sperm capacitation process and the apoptosis process during the maturity of sperm morphogenesis. T apoptosis-related genes in the mitochondria, including *Cyt-C, Bcl-2*, and *Bax*, disordered the gene expression, providing an exorbitant or depressed energy supply (18, 19), which might further interfere regularly with germ cell apoptosis and, therefore, inhibit semen quality (20).

For the present study, yellow-feathered breeder cocks were selected and differential levels of diet energy were provided to investigate the underlying mechanism of energy on semen quality and reproductive performances. We hypothesized that exorbitant or depressed energy provision inhibited the normal energy metabolism and further triggered the apoptosis process to lower spermatogenesis, which finally declined semen quality and reproductive performances.

## Materials and methods

Animal care and procedures followed The Chinese Guidelines for Animal Welfare, which was approved by the Animal Care and Use Committee of Jiangxi Agricultural University, Jiangxi, China, with the approval number JXAULL-20211009.

### Experimental design

A total of 90 yellow-feathered 13-week-old breeder cocks with the same age in days and similar body weight (1,437 ± 44.3,g) were randomly selected and divided into the low energy diet (LE), the moderate energy diet (ME), and the high energy diet (HE) treatment groups, respectively. Each treatment contained 6 replicates, with 5 birds in each replicate. All birds will receive a 7 days-long adaptation period followed by a 46-weeks feeding process. Chickens were raised in single cages in a fully enclosed house, received artificial ventilation, and 16 h of light per day. A total of 1 g of diet was provided to each bird at 6 a.m. and 3 p.m., respectively. The ingredients and nutritional levels of the diets are shown in Table 1. Other management systems and immunization procedures refer to the rules and management system of the experimental site.

**Table 1:** Ingredients and nutrient composition of experimental diets (DM basis %).

**Ingredients**	**Low energy**	**Moderate energy**	**High energy**
Corn	64.26	69.58	76.35
Soybean meal	18.12	18.08	16.68
Wheat bran	10.98	6.59	1.19
NaCl	0.31	0.32	0.33
Limestone	1.55	1.55	1.54
CaHPO4	1.77	1.80	1.85
Zeolite powder	2.34	1.40	1.31
Met	0.22	0.22	0.20
Lys	0.05	0.06	0.10
Thr	-	-	0.03
Trp	-	-	0.02
Choline chloride	0.18	0.18	0.18
Mineral premix^a^	0.20	0.20	0.20
Vitamin premix^b^	0.02	0.02	0.02
Total	100.00	100.00	100.00
Nutrients levels			
CP	14.00	14.00	14.00
ME /(MJ/kg)	11.30	11.72	12.14
NaCl	0.35	0.35	0.35
Ca	1.00	1.00	1.00
AP	0.42	0.42	0.42
Lys	0.70	0.70	0.70
Met	0.45	0.45	0.45
Met+Cys	0.70	0.70	0.70
Thr	0.58	0.58	0.58
Trp	0.18	0.18	0.18

a Mineral premix provided per kilogram of diet: Fe 60 mg, Cu 8.7 mg, Mn 90 mg, Zn 85 mg, I 0.5 mg, Se 0.4 mg.

b Vitamin premix provided per kilogram of diet: VA 12,000 IU, VD_3_ 3,500 IU, VE 45 mg, VK_3_ 2 mg, VB_1_3 mg, VB_2_12 mg, VB_6_10 mg, VB_12_ 30 mg, biotin 0.2 mg, calcium pantothenate 15 mg, nicotinic acid 30 mg, folic acid 1.5 mg.

### Sperm quality and fertility rate measurement

The parameters of semen quality, which mainly included the semen volume, pH, sperm density, sperm motility, and deformity rate, were further measured two times per week from 25 weeks old to 40 weeks old cocks before the mating test was initiated. Semen quality was measured according to the methods described by Sun et al. (21). To simply state, semen samples from each breeder cock were collected around 3 p.m. by the same skilled technician. The sperm collection process was done using the dorso-abdominal massage method, and care was taken in order not to contaminate the pooled semen through alien materials, such as feces and blood, which could reduce the quality of the collected semen (22). Semen volume was measured in a graduated collection tube immediately after the collection. Following, semen samples from each cock were diluted with 0.9% NaCl in a proportion of 1:100 and further used for evaluation of sperm motility, sperm viability, and sperm concentration with a computer-aided semen analysis system (ML-608JZII; Nanning Songjingtianlun Bio-technology Co., Ltd, Guangxi, China). Sperm motility was shown in the ten-point system while sperm viability was shown in the percentage form. The sperm deformity rate (calculated as the percentage of abnormal spermatozoa of the total (500) spermatozoa analyzed) was determined by *in vivo* staining with crystal violet and examined under light microscopy (Olympus, Tokyo, Japan) at 400× magnification.

Following, one breeder cock with the average semen quality in each replicate (a total of 18 roosters) was selected for fertility and hatchability measurement. The roosters were used as semen donors for artificial insemination (AI) in yellow-feathered layer hens of the same age, which were sexually rested for 4 weeks before the AI. The ratio of a rooster to hens was 1:10. AI was first performed on 2 consecutive days and two times again with 4 days in between. Eggs were marked and collected daily from day 2 to 14 after the first insemination and stored at a temperature of 18°C and relative humidity of 75% until incubation. Fertility for each rooster was calculated as the percentage of fertile eggs of the total number of set eggs. Hatchability of set eggs was calculated as the percentage of hatched eggs of the total number of set eggs. The healthy young rate was calculated as the percentage of healthy young chicken/total hatched eggs.

### Testis morphological measurement

All roosters used in the mating test were slaughtered at 50 weeks old for the collection of testicular tissue samples, weighed, quickly put into liquid nitrogen, and then transferred to a −80°C ultra-low temperature refrigerator for storage. Another part of testicular tissue was collected for section slicing and histomorphological observation. The left testis was cut into a small piece (1.0 cm in length, 1.0 cm in width, and 0.5 cm in height), followed by fixing in the formalin buffer for 48 h. All samples were further embedded in paraffin blocks, cut into sections of 5 μm thickness, and stained with the hematoxylin-eosin. Fifteen seminiferous tubules from each sample were selected for the measurement of seminiferous epithelium length (SEL), seminiferous tubule perimeter (STP), seminiferous tubule area (STA), and Johnsen score (23). All parameters involved in the development of the seminiferous tubule were measured using Image-Pro-Plus (version 6.0; Media Cybernetics, Inc. Maryland, USA) at 400× magnification.

### Spermatogenesis-related genes expression measurement

Spermatogenesis-related genes, which included the sry-related high mobility group box (SOX) gene family and the sperm-associated antigen (SPAG) gene family, were selected as the candidate genes for the measurement of expression through the reverse-transcriptional quantitative PCR (RT-PCR) method to determine the effects of energy treatment on spermatogenesis and sperm motility. The apoptosis-related genes in mitochondria, such as *Cyt-c, Bcl-2*, and *Bax*, were further measured to determine the underlying mechanism of energy supply on the development of testis and spermatogenesis. The total RNA of the testis sample was isolated from 30 to 50 mg of the sample using the TRIzol reagent (Invitrogen). Any residual gDNA and protein were removed with DNase I (TaKaRa, Japan) and the RNA clean kit (Tiangen, China), respectively. All sample RNAs were reverse transcribed into cDNA using the PrimeScript RT reagent Kit (Perfect Real Time) (TaKaRa, Japan) according to the instructions of the manufacturer. Quantitative PCR was performed using the ABI 7500 Real-time Detection System (Applied Biosystems) and SYBR Premix Ex Taq (Perfect Real Time) (TaKaRa). β-actin was amplified as an endogenous control. The primers of all candidate genes were designed using the Primer Premier 5.0 software and produced by Shenggong Biotech Co., Ltd. (Shanghai, China). All primers are listed in Table 2. The relative abundance of transcripts was calculated using the 2^−ΔΔ^CT method (24).

**Table 2:** Primers information of genes used in RT-qPCR.

**Gene name**	**Accession number**	**Primer sequence**	**Product size**	**Tm**
*SOX*5	NM_001004385.1	F:TGGGCTAAAGATGAACGG	187	54
		R:GCTTGTATTTGTAGTCTGGGT		
*SOX6*	XM_015286513.1	F:GTCATCTTTGGGTGATACAG	85	54
		R:TGAGCATAAATAGGCAGTC		
*SOX9*	NM_204281.1	F:CGATTACACCGAGCACCAGA	83	57
		R:AGGTGAATTGTTAGTAGAGGC		
*SOX13*	XM_015299129.1	F:AGCAGGTCAACATGCCGTAC	167	59
		R:CTTTAGCGTGGGAGGAGGAG		
*SPAG6*	NM_001199586.1	F:GAGATTATTGGAGGTATGAG	106	56
		R:CACCAGCAGCGTAAGTAG		
*SPAG16*	XM_421865.5	F:AGGGCTATAAAGCAGGGTG	129	58
		R:TGGCGAGAATAAGTAACATCAG		
*SPAG17*	XM_015294613.1	F:CGGCTTATCTATGAATGCT	165	54
		R:TGAAGTCGTTCCTCCTCTA		
*β-actin*	NM_001199586.1	F:GAGAAATTGTGCGTGACATCA	152	58
		R:CCTGAACCTCTCATTGCCA		

### Statistical analysis

The following parameters first received a normal distribution test using the SAS procedure “proc univariate data=test normal” (SAS Institute, Inc., Cary, NC, USA) and subsequently the one-way ANOVA S-N-K test: body weight, testis development parameters in each period, semen quality parameters, and testis morphometric development parameters. The relative abundance of transcripts was calculated using the 2^−ΔΔ^ method, followed by a one-way ANOVA S-N-K test to determine the discrepancy in the expression of genes among all treatments. The result was considered significant when a *p*-value was < 0.05 while a tendency was considered when 0.05 ≤ P < 0.10.

## Results

### Effects of differential energy level treatment on gonad development

Testes of breeder cocks were sampled during the pregenital, the fastigium, and the anaphase, respectively. All development information related to different periods of testes is shown in Table 3. The development of testes underwent a primary increment before they turned 40 weeks old, followed by a declining tendency. Testis did not maturely develop at 15 weeks old. The ME treatment showed superior body weight while the LE and HE treatments showed development performances of the testis, although not significant. Gonadosomatic index in the ME treatment significantly increased at 40 weeks old compared with the LE and HE treatments (*P* < 0.05). No other significant discrepancies were detected among all the three treatments.

**Table 3:** Effects of different energy levels on gonad development in different periods.

	**Items**	**Treatments**			**SEM**	***P*** **-value**
		**Low energy**	**Moderate energy**	**High energy**		
15-Weeks-old	Body weight	1,571	1578	1,576	38	0.612
	Left testis weight	4.48	4.39	4.37	1.06	0.343
	Right testis weight	4.62	4.51	4.57	1.28	0.447
	Total Testis weight	9.1	8.9	8.94	1.13	0.411
	Gonadosomatic index	0.6	0.57	0.57	0.14	0.251
40-Weeks-old	Body weight	2,303	2,320	2,420	2,76	0.667
	Left testis weight	19.12	22.08	20.08	5.53	0.396
	Right testis weight	18.45	20.58	19.58	4.61	0.414
	Testis weight	37.58	40.66	38.66	5.12	0.421
	Gonadosomatic index	1.63^b^	1.75^a^	1.59^b^	0.08	0.026
60-Weeks-old	Body weight	2,732	2,786	2,822	63.2	0.532
	Left testis weight	17.79	19.79	18.79	2.16	0.243
	Right testis weight	16.11	18.11	17.61	1.23	0.167
	Testis weight	34.71	37.91	36.41	2.02	0.089
	Gonadosomatic index	1.3	1.36	1.29	0.12	0.113

a, b Means in the same row parameters showed a significant difference.

### Semen quality and fertility rate

The results of semen quality are displayed in Table 4. Sperm motility showed a significant increment in the ME and HE treatments compared with the LE treatment (*P* < 0.05). Meanwhile, sperm deformity showed a downward trend in ME compared with the LE and HE. No significant differences were detected for other sperm quality parameters among all the three treatments.

**Table 4:** Effects of different energy levels on semen quality.

**Items**	**Treatments**			**SEM**	***P*** **-value**
	**Low energy**	**Moderate energy**	**High energy**		
Volume(ml)	0.49	0.52	0.47	0.04	0.134
pH	7.15	7.13	7.21	0.06	0.215
Density(10^9^/ml)	2.13	2.10	2.08	0.04	0.467
Motility (ten-point)	6.37^b^	6.87^a^	6.77^a^	0.31	0.048
Viability (%)	78.6	84.3	81.1	5.56	0.134
Deformity rate (%)	7.88	6.96	7.54	1.03	0.087

a, b Means in the same row parameters showed a significant difference, SEM, Standard error of the mean.

**Figure 1:**
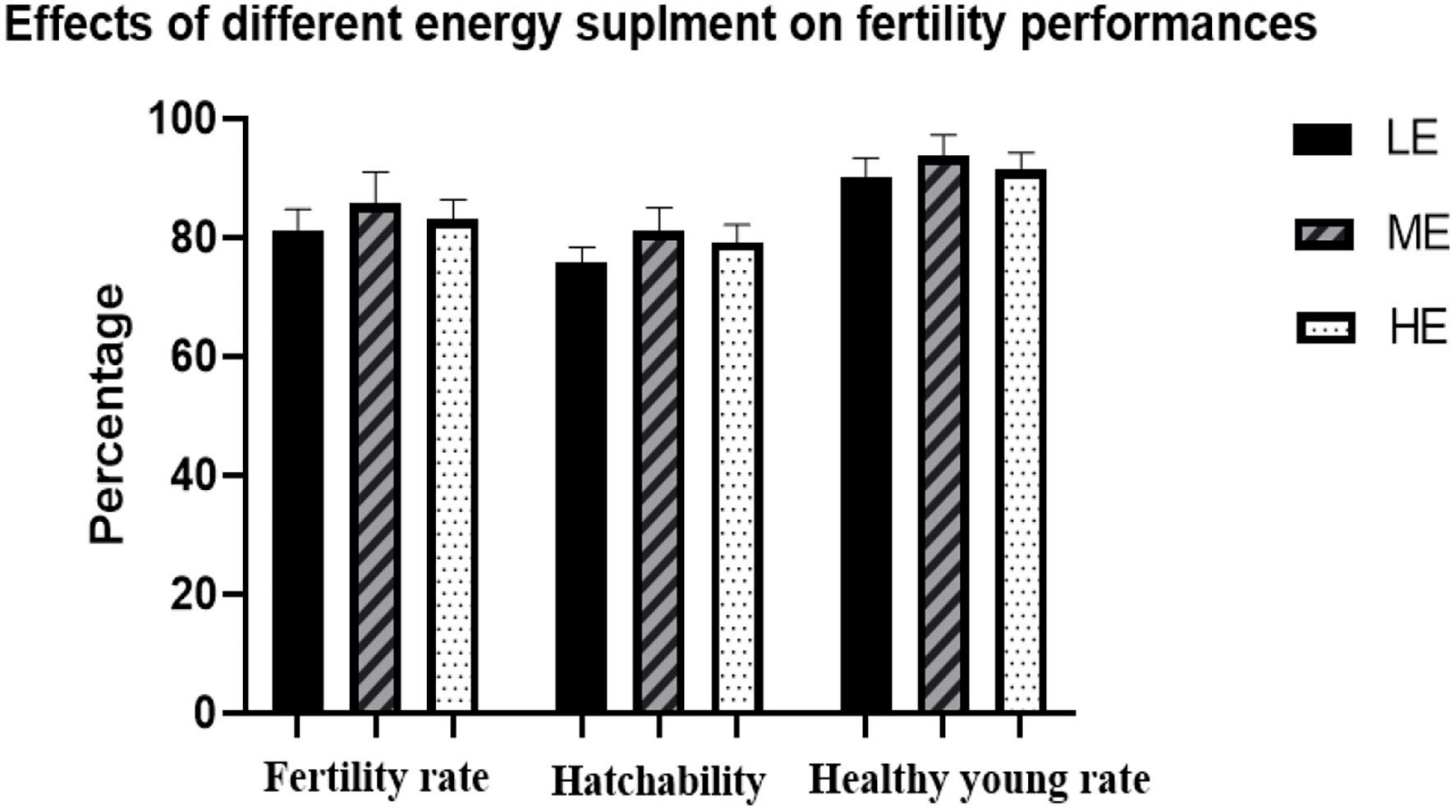
Effects of differential energy treatment on fertility rate, hatchability, and healthy young rate. LE, Low energy level; ME, Moderate energy level; HE, High energy level.

Subsequently, a mating test was further conducted to determine the effects of differential energy treatments on the fertility rate and hatchability. As seen in Figure 1, no significant differences were observed among all the three treatments on fertility rate, hatchability, and healthy young rate. ME slightly increased the fertility rate and healthy young rate.

### Testis morphology measurement

**Figure 2:**
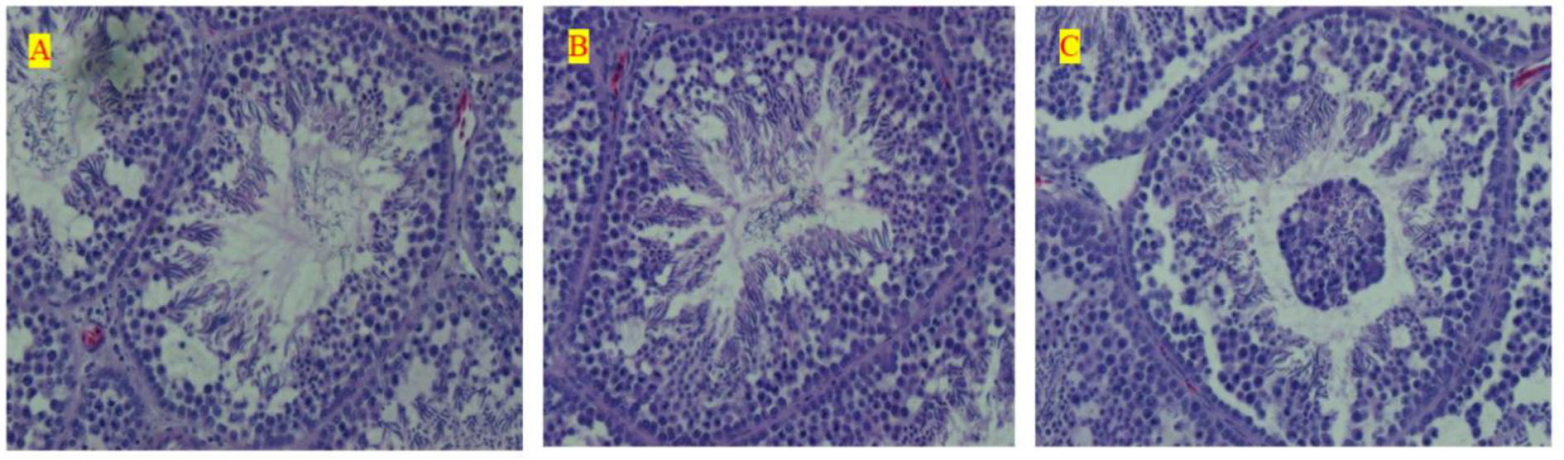
Effects of differential energy treatment on the morphological development of testes. **(A)** Seminiferous tubules of the effects of LE level treatment. **(B)** Seminiferous tubules of the effects of ME level treatment. **(C)** Seminiferous tubules of the effects of ME level treatment.

The measurement of testis morphology, which mainly included the gradient levels of spermatogenic cells in seminiferous tubules, is seen in Figure 2. Meanwhile, all morphological measurements based on the sections further received a discrepancy analysis among all the three treatments, and these results are seen in Table 5. The LE treatment remarkably inhibited the development of testis and postponed sexual maturity because of the significant decline in the SEL, STP, STA, and Johnsen Score than ME or HE at 15 weeks old. HE showed a significant increase in the development of testis before sexual maturity; however, it inhibited the testis development at the metaphase and the anaphase compared with LE and ME. Chickens under ME showed a preferable testis development performance at 40 and 60 weeks compared with the LE and HE. Particularly, the SEL and Johnsen Score significantly increased compared with the other two treatments. No significant changes were detected for other parameters during the raising period.

**Table 5:** Effects of different energy levels on morphometric development of testis and seminiferous tubules.

**Items**		**Low energy**	**Moderate energy**	**High energy**	**SEM**	***P*** **-value**
15-Week-old	SEL(μm)	10.31^c^	52.32^b^	61.77^a^	5.41	0.012
	STP (μm)	194.5^c^	228.1^b^	257.4^a^	18.37	0.043
	STA (10^4^ μm^2^)	2.35^c^	3.08^b^	3.77^a^	0.31	0.007
	Johnsen Score	2.34^c^	5.82 ^b^	6.41^a^	1.08	0.001
40-Week-old	SEL(μm)	146.4^b^	159.3^a^	151.7^ab^	13.24	0.031
	STP(μm)	596.7	628.4	613.4	36.71	0.141
	STA (10^4^ μm^2^)	19.95	21.38	20.77	2.31	0.203
	Johnsen Score	7.23^b^	8.82^a^	8.01^ab^	0.98	0.023
60-Week-old	SEL(μm)	125.4^b^	139.3^a^	131.7^ab^	13.24	0.031
	STP(μm)	576.7	620.4	603.4	46.7	0.241
	STA (10^4^ μm^2^)	17.95	20.38	19.77	3.41	0.273
	Johnsen Score	6.83^b^	8.12^a^	7.01^b^	0.78	0.043

a, b, c Means in the same row parameters showed a significant difference. SEL, seminiferous epithelium length; STP, seminiferous tubule perimeter; STA, seminiferous tubule area; SEM, standard errors of the mean.

### Spermatogenesis-related genes expression

**Figure 3:**
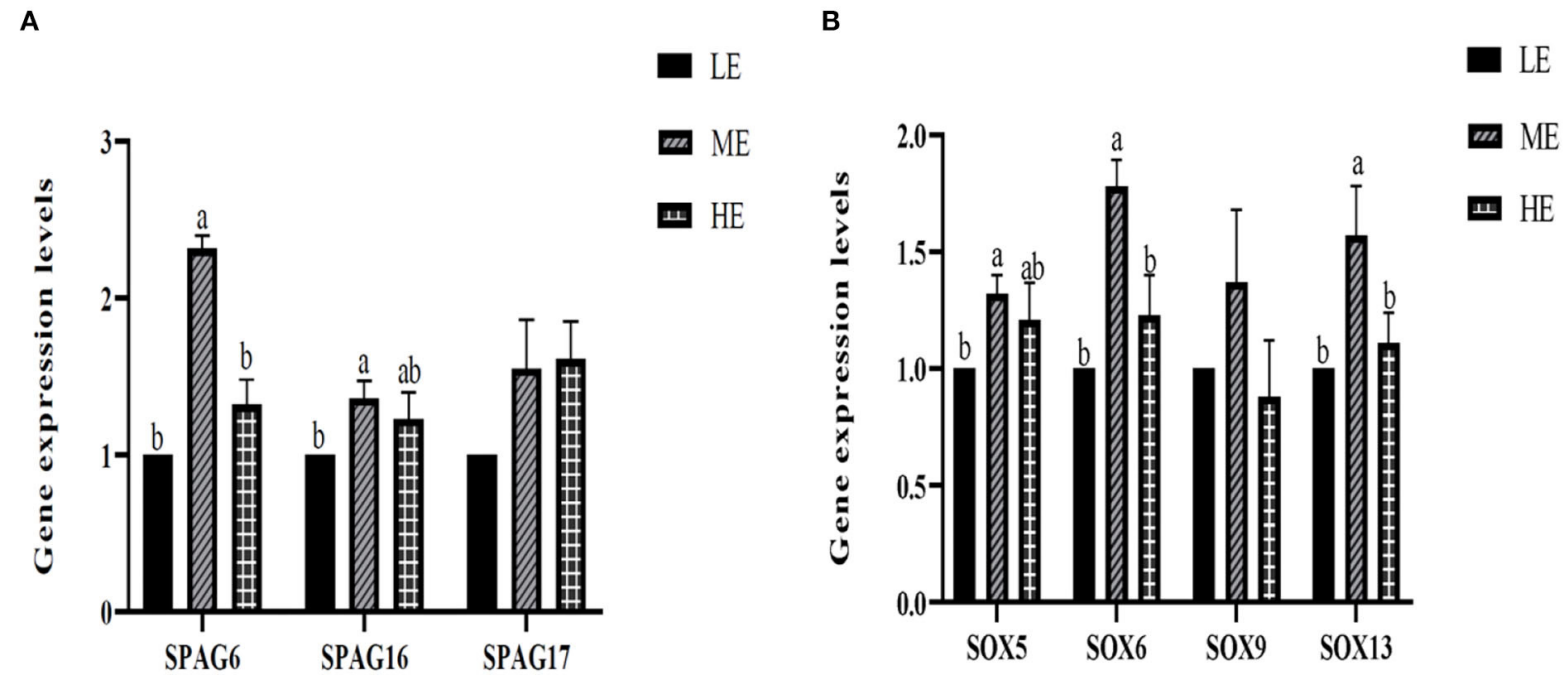
Effects of differential energy treatment on the expression of sperm motility-related genes. **(A)** Effects of differential energy treatment on the expression of the SPAG gene family. **(B)** Effects of differential energy treatment on the expression of the SOX gene family. LE, Low energy level; ME, Moderate energy level; HE, High energy level.

Spermatogenesis-related genes, which mainly included the *SPAG* gene family and the *SOX* gene family, were selected to determine the underlying mechanism of energy provision on spermatogenesis. Based on the results seen in Figure 3, *SPAG6* and *SPAG16* expressions were significantly increased in the ME treatment compared with the LE treatment, while *SPAG6* was also significantly upregulated in the ME treatment compared with HE. No significant changes were observed in *SPAG17* among all the three treatments. Further, the *SOX* gene family showed a similar expression trend with the *SPAG* gene family. ME significantly upregulated the expression of *SOX6* and *SOX13* compared with the other two treatments. Meanwhile, the *SOX5* gene expression significantly increased in ME compared with LE. No significant discrepancies were found in *SOX 9* among all the three treatments.

### Mitochondria apoptosis-related genes expression

**Figure 4:**
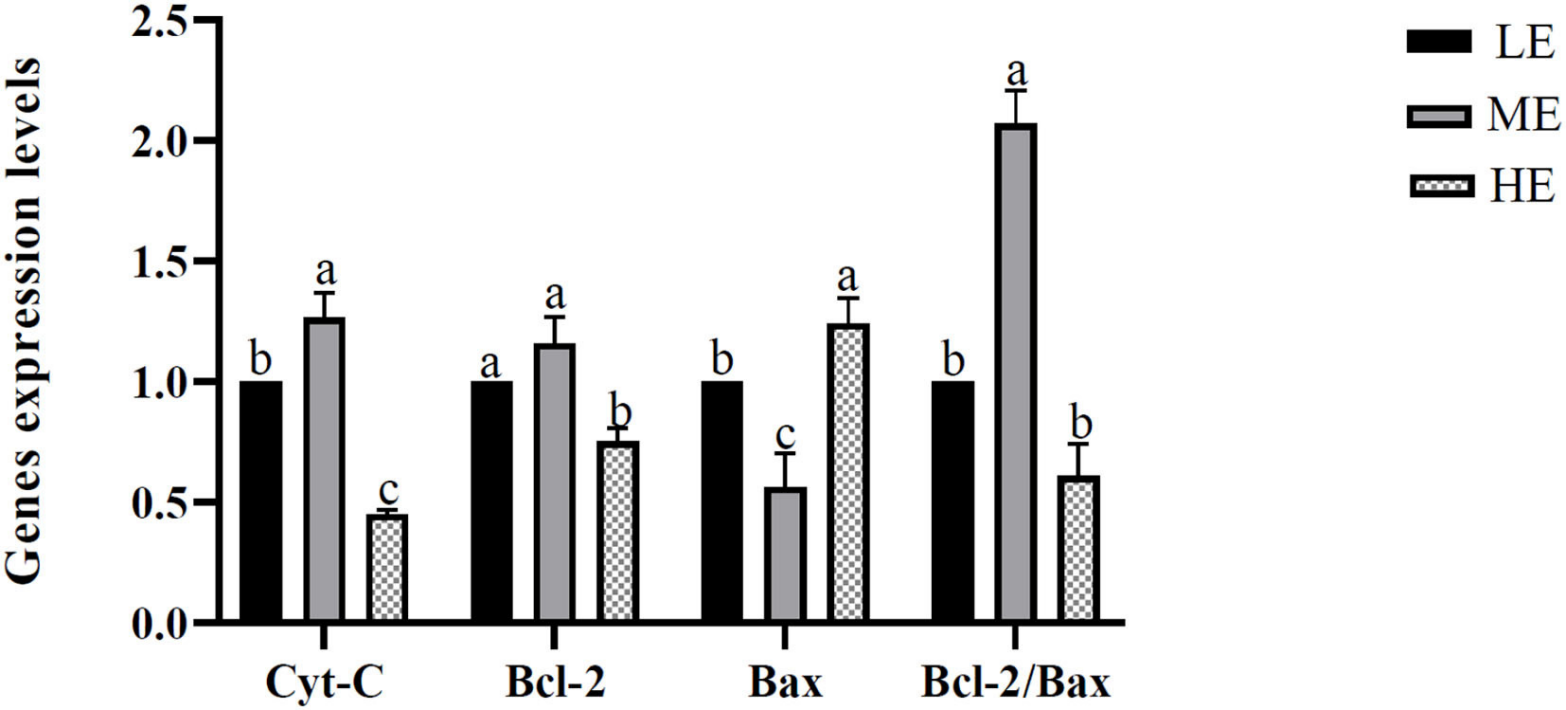
Effects of differential energy treatment on the expression of mitochondria energy metabolism-related genes. LE, Low energy level; ME, Moderate energy level; HE, High energy level.

The apoptosis-related genes of mitochondria, including the *Cyt-C, Bcl-2, Bax*, and *Bcl-2/ Bax*, were measured, and the results are seen in Figure 4. Expressions of *Cyt-C and Bcl-2* were significantly downregulated in the HE treatment compared with the LE and ME treatments. Meanwhile, *Cyt-C* was significantly upregulated in ME compared with LE. No significant discrepancy was observed between LE and ME on *Bcl-2* gene expression. *Bax* gene expression showed a complete reverse result compared with *Bcl-2*, which further causatively resulted in the significant increase of the ratio *Bcl-2/ Bax* in the ME treatment compared with the other two treatments. No significant discrepancy was observed in *Bcl-2/ Bax* ratio between LE and HE.

## Discussion

Dietary energy provision critically impacted poultry development and maturity during the chicken raising period. Exorbitant or depressed energy supply restricted a certain number of gene expressions, which, in turn, inhibited spermatogenesis and capacitation, thus causatively inducing the decline of semen quality and reproductive performances. Mitochondria performed an indispensable function in response to the energy alteration during spermatogenesis, which mainly included energy production and spermatogenic cell apoptosis (25). Therefore, the functions of mitochondria were especially concerned to determine the underlying mechanism of dietary energy provision on spermatogenesis and sperm motility of breeder cocks.

### Effects of differential energy provision on testis development and spermatogenesis

Testes for males harbored numbers of testicular somatic cells and germlines, which are essential for the propagation of the species (26). Similar to other vertebrates, the development and morphology of testes in birds differentiate during embryogenesis and further become condensed into seminiferous cords (27). However, unlike mammals, the testes of chickens are situated deep in the body cavity, which provided a more stabilized environment for testis development.

The development of testes is regulated by photoperiod, nutrition, and gene expression, which further modulated the hypothalamic–pituitary axis and causatively triggered gonad development. The considerable development of testes around the gonad maturity period was a huge energy consumption course (28), which may be the key factor that induced a significant decrease of testis weight in the LE treatment compared with the ME and HE treatments. Furthermore, more energy was required during spermatogenesis because spermatogenic cells triggered the differentiation process through both mitosis and meiosis, during which Sertoli and Leydig cells synchronously were developed to support the differentiation of germlines to synthesize mature sperms. The development of testis development was stagnated and degraded after the mating period or excessive artificial ejaculations. In this period, abundant energy was deposited into long-chain fatty acids, which depressed the development of testis and spermatogenesis and, therefore, causatively induced the decrease of gonad index and Johnsen Scores.

Moreover, energy provision may further regulate the development of testis and spermatogenesis by regulating the differentially expressed genes related to spermatogenesis (29). Since the members of the *SPAG* gene family including *SPAG6* and *SPAG16* and the *SOX* gene family including *SOX5, SOX6*, and *SOX13* were significantly upregulated in ME compared with LE, this finding was in line with Xinhong et al. (30) and Jiang et al. (31). The expression of *SPAG6* in testis is mainly expressed in spermatogenic cells, helping to promote the maturity of spermatogenesis (12). Increased SPAGs expression promoted sperm maturity, therefore increasing the Johnsen Score and sperm motility. In addition, *the SOX* gene family also executed critical effects during testis formation, spermatogenesis, and capacitation, for these genes were expressed in peak in around 15-week-old roosters to promote the development of testis (32–34), which may further help increase the fertility rate.

### Effects of differential energy provision on fertility rate

Fertility rate provided the initial direct indicator to evaluate the reproductive performance of breeder cocks, which was mainly impacted by semen quality, especially sperm motility and deformity and the physiological condition of layer hens (35). In contrast, semen quality significantly correlated with the fertility rate. In our present study, although not significant, the fertility rate in ME showed an obvious increment when compared with LE. The underlying mechanism of energy treatment on fertility rate might be attributed to the following reasons.

The activity of mitochondrial bioprocess remarkably regulated spermatogenesis and played diverse roles in the whole process of gamete production (36). Spermatozoa derive energy from the oxidative metabolism to perform normal physiological functions, although they can survive also by glycolytic energy. Abnormal energy transmission in the mitochondria induced infertile sperm motility, which may further cause a decrease in fertility (37). The regulatory effects of the mitochondria on spermatogenesis might be attributed to the degenerative processes of germ cells to ensure the high quality of spermatozoa and to further the regulatory effects on intrinsic apoptosis (38). Intrinsic apoptosis mediated by mitochondrial signaling was shown to play a crucial role in the modulation of apoptosis during the primordial germ cells (PGCs) maturity to avoid anomalous migration into the gonads. Apoptosis-related genes including the *Cyt-C, Bax*, and *Bcl-2* were expressed abnormally in both the LE and HE treatments, which may restrict the formal maturity of sperms, thus decreasing the fertility rate (39).

In addition, spermatogenic-related genes modulated sperm motility and further impacted the fertility rate. The gene family of sperm flagellar axoneme protein interacted with each other to regulate the motility of flagella and played a decisive role in sperm motility (40), which thereby upregulated the expression of *SPAG* gene members to help increase the fertility rate. Moreover, the expression levels of *SOX5, SOX6, SOX9*, and *SOX13* in the testes of normal roosters were significantly higher than those of asthenozoospermic roosters. Meanwhile, the *SOX* gene family significantly promoted sperm flagellar motility (41). When the expression of the *SOX* gene family was downregulated, the flagellar structure changed, and spermatogenesis, morphology, and motility declined. Meanwhile, the *SOX* genes interacted with the promoter regions of sperm motility candidate genes such as *CATSPER1* (42). The upregulation of the *SOX* genes may further promote the expression of *CATSPER1* gene, thereby increasing sperm motility and fertility rate.

In summary, energy provision to yellow-feathered breeder cocks played a critical role in semen quality and reproductive performances. ME provision stimulated normal energy metabolism and further triggered the normal apoptosis process for spermatogenesis, which finally increased semen quality and reproductive performances.

## Data availability statement

The original contributions presented in the study are included in the article/supplementary material, further inquiries can be directed to the corresponding author.

## Ethics statement

The animal study was reviewed and approved by JXAULL-20211009. Written informed consent was obtained from the owners for the participation of their animals in this study.

## Author contributions

FX and JS designed the study. YL, ZLv, and JZ conducted the experiment. SX, LZ, and ZLi contributed to parameter measurement and data analysis. All authors contributed to the article and approved the submitted version. All authors carefully read and are accountable for all aspects of the work.

## Funding

This research was funded by the Natural Science Foundation of Jiangsu Province (BK20211121). It was also funded by the JBGS Project of Seed Industry Revitalization in Jiangsu Province (JBGS [2021] 107) and the Special Fund for Independent Innovation of Agricultural Science and Technology in Jiangsu Province of China [CZ (20) 3001].

## Conflict of interest

The authors declare that the research was conducted in the absence of any commercial or financial relationships that could be construed as a potential conflict of interest.

## Publisher's note

All claims expressed in this article are solely those of the authors and do not necessarily represent those of their affiliated organizations, or those of the publisher, the editors and the reviewers. Any product that may be evaluated in this article, or claim that may be made by its manufacturer, is not guaranteed or endorsed by the publisher.
